# Indirect Short- and Long-Term Effects of Aboveground Invertebrate and Vertebrate Herbivores on Soil Microarthropod Communities

**DOI:** 10.1371/journal.pone.0118679

**Published:** 2015-03-04

**Authors:** Martijn L. Vandegehuchte, Ursina Raschein, Martin Schütz, Dariusz J. Gwiazdowicz, Anita C. Risch

**Affiliations:** 1 Research Unit Community Ecology, Swiss Federal Institute for Forest, Snow and Landscape Research, Birmensdorf, Switzerland; 2 Institute of Evolutionary Biology and Environmental Studies, University of Zürich, Zürich, Switzerland; 3 Faculty of Forestry, Poznań University of Life Sciences, Poznań, Poland; Helmholtz Centre for Environmental Research (UFZ), GERMANY

## Abstract

Recognition is growing that besides ungulates, small vertebrate and invertebrate herbivores are important drivers of grassland functioning. Even though soil microarthropods play key roles in several soil processes, effects of herbivores—especially those of smaller body size—on their communities are not well understood. Therefore, we progressively excluded large, medium and small vertebrate and invertebrate herbivores for three growing seasons using size-selective fences in two vegetation types in subalpine grasslands; short-grass and tall-grass vegetation generated by high and low historical levels of ungulate grazing. Herbivore exclusions generally had few effects on microarthropod communities, but exclusion of all herbivore groups resulted in decreased total springtail and Poduromorpha richness compared with exclusion of only ungulates and medium-sized mammals, regardless of vegetation type. The tall-grass vegetation had a higher total springtail richness and mesostigmatid mite abundance than the short-grass vegetation and a different oribatid mite community composition. Although several biotic and abiotic variables differed between the exclusion treatments and vegetation types, effects on soil microarthropods were best explained by differences in nutrient and fibre content of the previous year’s vegetation, a proxy for litter quality, and to a lesser extent soil temperature. After three growing seasons, smaller herbivores had a stronger impact on these functionally important soil microarthropod communities than large herbivores. Over longer time-scales, however, large grazers created two different vegetation types and thereby influenced microarthropod communities bottom-up, e.g. by altering resource quality. Hence, both short- and long-term consequences of herbivory affected the structure of the soil microarthropod community.

## Introduction

Grazing is widely recognised as one of the main ecosystem drivers in grasslands [1]. A large body of literature has provided proof of the manifold influences of large grazers, mainly ungulates, on the structure and functioning of grasslands [2]. Recently, it is becoming increasingly clear that the functionally different smaller vertebrate [3, 4] and invertebrate [5] herbivores also play important roles in the ecology of these ecosystems. Like large herbivores, medium-sized and small herbivores can lower plant abundance through consumption, lower or enhance plant productivity through changes in nutrient cycling, increase plant biomass through changes in competitive interactions among plants or alter plant diversity [3–6]. However, large grazers are assumed to exert larger effects on plant populations than insects, as they consume more plant tissue [7]. Yet, burrowing mammals create patches of disturbed soil and short vegetation and thus increase habitat heterogeneity [8]. Smaller herbivores tend to return nutrients (faeces, urine) to the system more evenly distributed compared with large grazers [9].

It has also been shown that the effects of large grazers depend on the productivity of the ecosystem [1, 10, 11], while those of invertebrate herbivores do not seem to be related to productivity [12]. Regardless of body size, herbivore-induced changes in grassland ecosystem properties have been shown to affect components of grassland biodiversity other than plants, of which arthropods are one of the largest [13]. While the impact of grazers on aboveground arthropod communities has been documented quite extensively, studies of their effects on belowground arthropod communities are fewer and are mostly restricted to domestic grazers in agroecosystems [14–17]. These effects of aboveground herbivores are potentially important since soil microarthropods are known to play crucial roles in ecosystem functions such as carbon and nitrogen mineralisation, plant growth and organic matter decomposition [18–20].

The few studies on soil microarthropod responses to mammalian grazers report negative, positive or neutral reactions. Negative effects are generally related to disturbance of the soil through trampling or burrowing [17, 21, 22]. Positive effects are generally attributed to increased microbial biomass [15], caused by increased root exudation and the labile nutrients in dung and urine [11]. Some litter microarthropods, moreover, have been shown to react positively to grazing because of the increased soil temperature as a result of lower plant cover providing less shade [16]. In addition to short-term effects, e.g. produced by trampling or burrowing, grazing by mammals can cause profound long-term changes in the vegetation [1, 2], which have the potential to alter soil microarthropod communities [14]. Compared with what we know about how large ungulates impact soil microarthropod communities, literature on the impact of aboveground invertebrate grazers is limited. Yet, it has been reported that aboveground invertebrate herbivory can decrease leaf litter quality through induced plant defences [23]. This is not unique to invertebrate herbivores, but as invertebrates, especially sapsuckers, do not remove as much plant tissue as large grazers, potentially more defence compounds reach the soil when dead plant tissue decomposes. Invertebrate herbivory can also accelerate leaf senescence, which reduces nutrient resorption and increases litter nutrient amounts [6]. Furthermore, frass, cadavers and nutrient leaching from damaged leaves can increase the levels of nutrients reaching the soil [6]. Thus, depending on the mechanism, aboveground invertebrate herbivores could increase or decrease the quantity and quality of substrates in the soil, which in turn could influence root growth as well as biomass of different groups of microbes. Such changes could have a direct positive or negative influence on soil microarthropod abundance and diversity, as these seem to be largely controlled by bottom-up forces, mainly by resources (e.g. microbial biomass and root tissue [24, 25]).

The ecological importance of soil microarthropods notwithstanding, no study has, to our knowledge, simultaneously assessed how functionally different vertebrate and invertebrate herbivores alter their communities. We conducted a field experiment that uses size-selective fences to progressively exclude large, medium and small vertebrate as well as invertebrate herbivores for three consecutive summers in two types of subalpine grassland in the Swiss National Park (SNP); productive short-grass and less productive tall-grass vegetation. Both vegetation types are the result of high or low wild ungulate grazing intensities respectively since roughly 70 years. We tested how the herbivore assemblages of different body size and the vegetation type affected the community composition, abundance and richness of soil microarthropods.

We test the following hypotheses: H1) The exclusion of ungulates and medium-sized mammals would benefit soil microarthropod abundance by eliminating trampling and burrowing disturbance, outweighing possible negative effects of lowered microbial biomass due to decreased nutrient returns. H2) Progressively excluding aboveground herbivores concurrently increases aboveground plant biomass, leading to increased shading and lower soil temperatures, which negatively affect soil microarthropods, thus partly offsetting the positive effects of eliminating disturbance. H3) These effects are more pronounced in the short-grass vegetation where the background levels of disturbance are higher and where increases in aboveground biomass would be steeper. H4) The exclusion of invertebrate herbivores negatively affects soil microarthropod communities as in grassland ecosystems the negative effect of decreased nutrient input to soil likely exceeds the benefit of reduced defence chemicals in plant litter. H5) The tall-grass vegetation supports higher abundances of soil microarthropods than the short-grass vegetation due to its higher root and soil microbial biomass providing a larger food source as a result of the long-term differences in ungulate grazing intensities. We expected changes in soil microarthropod diversity to concur with changes in abundance.

## Materials and Methods

### Study area

The SNP is situated in the south-eastern part of Switzerland (6°40’N, 10°15’E). It covers an area of 170 km^2^ of which 86 km^2^ is vegetated. Forests (mainly pine) cover 50 km^2^, alpine grasslands 33 km^2^ and subalpine grasslands 3 km^2^. Elevations range from 1400 to 3174 m asl. Mean annual precipitation and temperature are 871 ± 156 mm and 0.6 ± 0.6°C (mean ± SD) measured at the park’s weather station in Buffalora (1980 m asl) between 1960 and 2009 [26]. Since the establishment of the SNP in 1914, regulations have minimised human interference with nature. Hunting, camping, fishing and dogs are not allowed and visitors have to stay on marked trails. A field permit was issued by the Swiss Academy of Natural Sciences—Research Commission of the Swiss National Park.

Large patches of two vegetation types can be distinguished in the subalpine grasslands. Short-grass vegetation is dominated by lawn grasses and has an average height of 2–5 cm. Tussock graminoids are dominant in the tall-grass vegetation, which has an average height of 20 cm [27]. Before the creation of the SNP (14^th^ century until 1914), cattle and sheep grazed on the subalpine grasslands. Short-grass vegetation developed in parts of the grasslands where cattle and sheep used to rest (around stables and huts) and which therefore received high nutrient inputs. These parts are still preferentially grazed by wild ungulates today, which re-immigrated into the park after its foundation [27, 28]. Tall-grass vegetation developed where domestic ungulates used to graze, but did not rest.

We distinguished herbivores of four body size categories inhabiting the subalpine grasslands in the SNP: large (red deer (*Cervus elaphus* L.) and chamois (*Rupicapra rupicapra* L.), 30–150 kg), medium (alpine marmot (*Marmota marmota* L.) and mountain hare (*Lepus timidus* L.), 3–6 kg), and small vertebrates (small rodents: e.g. *Clethrionomys* spp., *Microtus* spp., *Apodemus* spp., 30–100 g) as well as invertebrates (e.g. Orthoptera, Lepidoptera, Auchenorrhyncha, Aphidoidea, < 5 g).

### Experimental design

The construction of exclosures has been described in detail in Risch et al. [29] and Haynes et al. [30]. Six subalpine grasslands were selected throughout the SNP, in which hierarchical exclosure setups were erected, spanning elevations from 1975 to 2299 m asl. The three larger grasslands had four exclosure setups each (two in short-grass and two in tall-grass vegetation) while the three smaller grasslands had two setups each (one in each vegetation type), for a total of 18 exclosure setups, 9 in short-grass and 9 in tall-grass vegetation. These setups were installed in early June 2009, immediately after snowmelt. Each exclosure setup consisted of 5 plots, each of which received a different herbivore exclusion treatment. The main fence was constructed of wooden posts with electric equestrian tape mounted at 0.7, 0.95, 1.2, 1.5, and 2.1 m above the ground and connected to a solar charged battery. Equestrian tape not connected to the power source was mounted at 0.5 m to help exclude deer and chamois but to ensure that smaller herbivores could enter safely. Within this fence, 4 plots of 2 × 3 m were delineated and randomly assigned to one of four treatments. One plot was left unfenced, so with the exception of ungulates, all other herbivores had access (“Marmot/Mouse/Invertebrate”). A second plot was fenced with electric sheep fence to further exclude marmots and hares, but still allow access by small rodents and invertebrates (“Mouse/Invertebrate”). A third plot was fenced with a metal wire mesh to exclude all vertebrate herbivores, but still enable access by invertebrates (“Invertebrate”). A fourth plot was enclosed by a fence and a roof, consisting of metal mosquito netting, to exclude aboveground invertebrates (“None”). A biocompatible insecticide was applied in this plot when necessary to eliminate invertebrates that might have hatched from the soil or entered when the fence was opened for data collection. A 1 m wide buffer strip separated these four plots from each other. A fifth plot, located at least 5 m from the outer fence, served as an unfenced control plot where all types of herbivores had access (“All”). We realise that our treatments did not only exclude herbivores, but also other animals of the same body size. We defined the effect of an excluded community composed of herbivores, predators and other functional groups as a net level of herbivory, caused by a certain number of herbivores, regulated among other things by predation, parasitism and competition. Note that the “None” plots only excluded aboveground invertebrates. Soil dwelling organisms still had access to all plots.

At six of the 18 exclosure setups (one in each of the six grasslands) an additional fence construction was set up at 15 m from the main exclosure setup to assess the effect of the roof of the “None” exclosures on microclimatic parameters (“Microclimate control”; for more detail see [29, 30]). Measurements confirmed that except for a decrease in incoming UV light, the mosquito netting did neither alter any microclimatic variables nor aboveground plant biomass, so that changes observed in the “None” plots were attributed to the exclusion of the invertebrates [29, 30]. All fences were dismantled in autumn to protect them from snow pressure and avalanches and remounted the next spring immediately after snowmelt.

Marmot counts were performed twice each summer and confirmed their presence at all sites. Small rodents were not quantified at the sites and the different herbivores were not counted within the exclosures. However, game cameras revealed that mice entered some “Mouse/Invertebrate” plots and that marmots, hares and mice entered the “Marmot/Mouse/Invertebrate” plots.

### Soil microarthropod sampling and identification

One soil core (5 cm diameter, 10 cm depth) was taken monthly in all five treatment plots at each of the 18 exclosure setups from late June to late August 2011, for a total of 270 samples. The soil corer (AMS Samplers, American Falls, ID, USA) was fitted with a plastic liner to ensure collection of an undisturbed sample. The lined core was removed from the corer, sealed on both ends with cling film and transferred to a cooler. All plots were sampled within a time span of three days, and extractions were initiated in the evening of the sampling day, using a high-gradient Tullgren funnel designed after Crossley and Blair [31]. The bottom of each liner was covered with cheesecloth to keep soil particles from falling into the collection vial. Samples were kept in the extractor for four days, and soil arthropods were collected in 95% ethanol. Arthropods were enumerated in the laboratory using a stereomicroscope and identified to the lowest taxonomic level possible with the aid of a compound microscope. For identification under the microscope, the arthropods were macerated with lactic acid (80%) and then either mounted on microscope slides or identified on cavity slides in lactic acid. Collembola and Oribatida were identified to species level whenever possible, otherwise to genus, family or morphospecies. Prostigmata were categorised into morphospecies. Mesostigmata were classified into genera when possible, otherwise into cohorts or morphospecies. Other soil arthropods (e.g. millipedes, insect larvae) were counted, but not considered in this study.

### Sampling of vegetation and soil properties

Vegetation and soil properties were measured in each plot during the 2011 growing season. Shoot biomass per m^2^ was estimated non-destructively on a 1 × 1 m subplot in each plot at peak biomass using the canopy intercept method [32]. Five soil samples (2.2 cm diameter, 10 cm depth; Giddings Machine Company, Windsor, CO, USA) for determination of root biomass were taken at random in another subplot in early September from two 10 × 100 cm strips after removing the vegetation. The roots were manually separated from the soil and the average root biomass of the five samples was used to calculate the root biomass per m^2^ for each plot (for more details see [29]).

In addition, six soil samples were randomly collected from the two strips with a 5 cm diameter by 10 cm soil corer (AMS Samplers, American Falls, ID, USA). The organic soil (usually the top 1 to 5 cm) was separated from the mineral soil. Mineral soil samples were combined per three into two composite samples and the six organic soil samples were pooled. They were immediately put on ice, transported to the laboratory, passed through a 2 mm sieve and stored at 4°C. On one of the composite mineral soil samples, microbial biomass carbon (MBC) was determined with the substrate-induced method of Anderson and Domsch [33]. The other composite mineral soil sample and the composite organic soil sample were analysed for carbon (C) and nitrogen (N) concentration (Leco TruSpec Analyzer, Leco, St. Joseph, MI, USA), and C/N ratios were calculated. Soil moisture and soil temperature were measured every fortnight between 0900 and 1700 h from late May until late August 2011 (8 times in total). Soil moisture was measured at five random points in each plot with a Field-Scout TDR-100 (time domain reflectometer; Spectrum Technologies, Plainfield IL, USA) for the 0 to 10 cm soil depth and averaged per plot. Soil temperature was measured once per plot for the 0 to 10 cm depth with waterproof digital pocket thermometers (Barnstead International, Dubuque IA, USA). Soil moisture and temperature values were subsequently averaged per plot across the 8 measurement dates for further analyses. We measured shoot C and N content as well as neutral detergent fibre (NDF) content of vegetation clipped in July 2010 (peak biomass) and September 2010 (start of senescence). We used last year’s vegetation quality as proxy for the quality of the litter incorporated into the soil the current year. Vegetation was removed from two 10 × 100 cm strips in each treatment plot, combined per plot (n = 90), dried, ground (Pulverisette 16, Fritsch, Idar-Oberstein, Germany), and passed through a 0.5 mm sieve. Shoot N, C (Leco TruSpec Analyzer, Leco, St. Joseph, MI, USA) and NDF (Fibre Analyzer 200/220, ANKOM Technology, NY, USA) concentrations of selected samples were measured. Shoot C, N and NDF were subsequently estimated for all samples from models previously established for the SNP relating Fourier transform-near infrared reflectance (FT-NIR) spectra to measured values of C, N and NDF using a MPA multi-purpose FT-NIR spectrometer (Bruker Optics, Switzerland).

### Statistical analyses

The experiment was analysed as a split-plot design. Each of the 18 exclosure setups acted as a whole plot, split into 5 different herbivore exclusion treatment plots, i.e. subplots. Vegetation type was the whole-plot factor, with exclosure setups belonging to levels “short-grass” or “tall-grass”. Herbivore exclusion treatment was the subplot factor, with levels “All”, “Marmot/Mouse/Invertebrate”, “Mouse/Invertebrate”, “Invertebrate” and “None” each assigned to a different subplot of each exclosure setup. The exclosure setups (whole plots) were aggregated within grasslands, which served as blocks. We used uni- and multivariate distance-based permutational ANOVA [34] with vegetation type, treatment and their interaction as fixed effects and grassland and exclosure setup as random effects, permuting residuals under a reduced model. In the univariate case the analyses are similar to classical split-plot ANOVA. However, as they are based on permutation methods, there is no assumption of normality, which lends them well to the analysis of e.g. count data. Note that the assumption of homogeneous variances remains. Using a single methodology furthermore makes results readily comparable between uni- and multivariate analyses.

Dependent variables in the univariate analyses were taxon richness (further referred to as richness) and abundance of the following taxonomic groups of soil microarthropods: Acari, suborder Prostigmata (Acari, order Trombidiformes), suborder Monogynaspida (Acari, order Mesostigmata), suborder Oribatida (Acari, order Sarcoptiformes), Collembola, order Entomobryomorpha (Collembola), order Poduromorpha (Collembola); as well as the vegetation (root and shoot biomass, July and September vegetation NDF, C and N content and C/N ratio) and soil (C and N content and C/N ratio of organic and mineral soil; soil temperature, moisture and microbial biomass C) characteristics. We studied the taxonomic groups mentioned above, as they are generally characterised by functional differences (e.g. in food type, activity, body structure or lifespan; see discussion). Juvenile microarthropods were omitted from all analyses, as we focus on relative differences in microarthropod communities between vegetation types and herbivore exclusion treatments, not on absolute abundances of different microarthropod groups. “Acari” and “Collembola”, refer to the total of all adult individuals throughout the text. Abundances of the different microarthropod taxa were summed across the three sampling dates for analyses. Richness was calculated for each microarthropod group as the number of taxa that occurred in a plot on at least one of the three sampling dates. Abundance and richness of the orders Neelipleona (Collembola) and Symphypleona (Collembola) were too low to analyse separately, but they are included in the analyses of Collembola. The multivariate analyses (distance-based permutational MANOVA) were used to assess differences in the community composition of the microarthropod taxonomic groups (sample by taxon matrix). All univariate analyses were based on Euclidean distances between samples, multivariate analyses on Bray-Curtis dissimilarities between samples. In case of undefined Bray-Curtis coefficients (two samples with zero observations), the zero-adjusted Bray-Curtis dissimilarity measure was used [35]. In the case of a significant (M)ANOVA effect, we additionally performed a permutational test of dispersion (uni- or multivariate) for that variable. If the dispersion test was significant, we transformed the dependent variables until the dispersions were homogeneous (*P* > 0.05) or the (M)ANOVA effect was no longer significant. Community matrices of Acari, Prostigmata and Monogynaspida were square root transformed. Monogynaspida abundance was fourth root transformed. Post hoc pairwise comparisons between levels of a significant factor were made using permutational t-tests. Post hoc *P*-values were corrected for multiple comparisons using the Benjamini-Hochberg false discovery rate (FDR) [36]. In the case of significant vegetation type by treatment interactions, we performed pairwise comparisons between treatments within each vegetation type.

To assess mechanisms by which herbivore exclusions and vegetation type affected soil microarthropod communities, we selected the initial models with significant effects of vegetation type or exclusion treatments on soil microarthropods and included plant or soil characteristics as a covariate. If a vegetation type or treatment effect on soil microarthropods is entirely due to changes in a certain plant or soil characteristic, then any significant effect of these factors in the initial model should become non-significant after taking into account the relationship with this plant or soil characteristic. If, however, the initial vegetation type or treatment effect remains significant, this implies an effect independent of the relationship with the plant or soil characteristic. For each covariate, a separate model was run, sequentially fitting grassland, vegetation type, exclosure setup, treatment and vegetation type × treatment. In initial models with a significant vegetation type effect the covariate was introduced at the whole plot level and was fitted after the random grassland effect. In initial models with a significant treatment effect the covariate was introduced at the subplot level by fitting it after the random exclosure setup effect. All tests used 99 999 permutations. Monte Carlo *P*-values were obtained if the number of unique permutations fell below 100. All reported F-values are pseudo-F values as per Anderson et al. [34].

Because the springtail *Folsomia quadrioculata* (Tullberg, 1871) is actually a litter-dwelling species and was consistently absent in the “None” plots, we cannot rule out the possibility that it was directly excluded by our “None” treatment, instead of being influenced by the absence of aboveground animals. It was therefore omitted from the analyses. All the other observed microarthropods were considered soil-dwelling and hence not directly affected by the aboveground “None” treatment, which was indeed confirmed by our data.

To represent multivariate analyses graphically, we performed Nonmetric Multidimensional Scaling (NMS) ordinations based on 50 restarts and a stability criterion of 0.001. To visualise effects of vegetation type, the ordination was performed on the centroids of the 18 exclosure setups.

## Results

### Effects of herbivore exclusion treatment and vegetation type on soil microarthropod taxonomic groups

**Fig 1:**
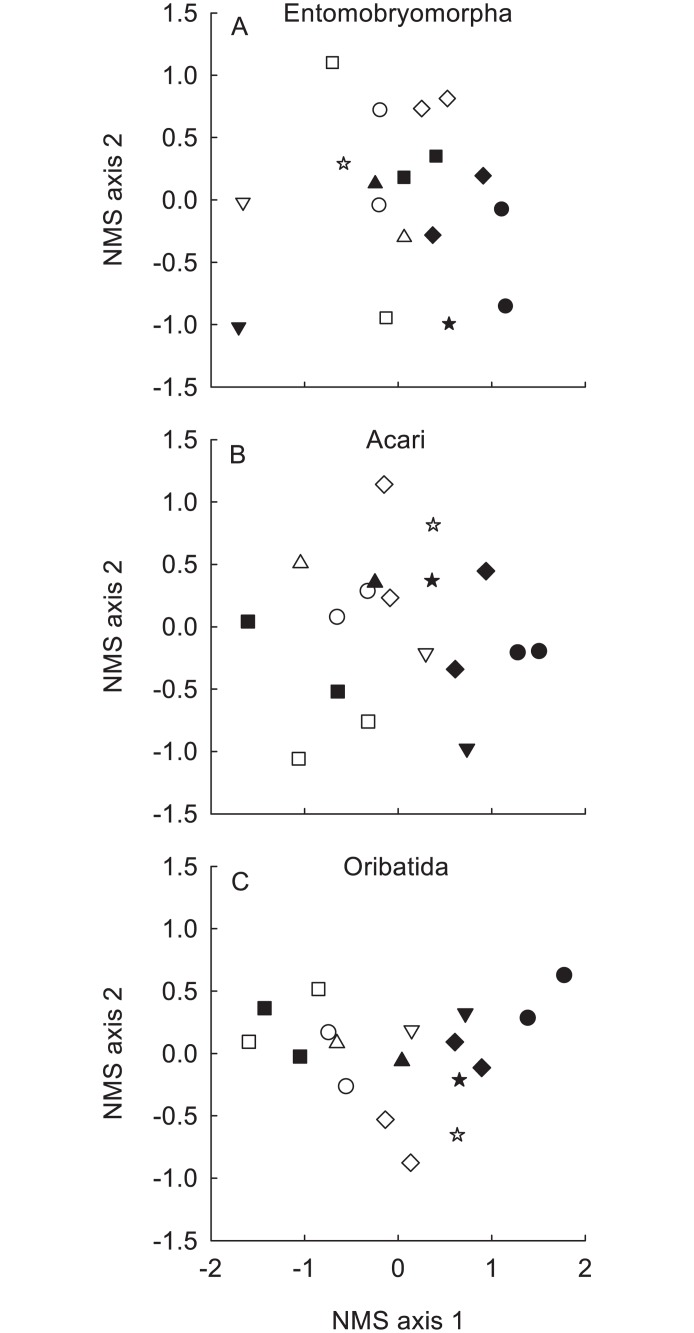
Nonmetric Multidimensional Scaling (NMS) ordination plot of soil microarthropod communities. To avoid plotting 90 points, for clarity, each point represents the centroid of one of the 18 exclosure setups, combining the 5 herbivore exclusion treatments, as these had no effect. Same symbols depict tall- and short-grass sites from same grasslands. White symbols—short-grass vegetation; black symbols—tall-grass vegetation. A) Entomobryomorpha. B) Acari. C) Oribatida.

We extracted a total of 5489 adult mites (358 Monogynaspida, 1433 Oribatida and 3698 Prostigmata) and 1963 adult springtails (963 Poduromorpha, 912 Entomobryomorpha, 19 Neelipleona and 69 Symphypleona) and distinguished 12 springtail and 53 mite taxa in total (S1 Table). The community composition of Acari, Entomobryomorpha and Oribatida significantly differed between vegetation types (Table 1, Fig. 1a,b,c). Fig 1. shows some overlap between the two vegetation types (black and white symbols), and significant separation between the grasslands (different shapes), but within each grassland (same shapes) there was generally a consistent shift between short- and tall grass vegetation (black and white symbols). None of the investigated soil microarthropod groups’ community composition showed a significant response to the herbivore exclusion treatments (Table 1).

**Table 1:** Results of the permutational (M)ANOVAs testing the effects of the vegetation type (Vegetation), herbivore exclosure treatment (Treatment) and their interaction (Veg × Treat) on the richness, community composition and abundance of different taxonomic groups of soil mites and springtails as a split-plot with grassland (Grassland) and exclosure setup (Site) as random effects.

		Richness			Community Composition			Abundance		
Source	df	F	p	perms	F	p	perms	F	p	perms
Collembola										
Grassland	5,11	0.94723	0.4904	95887	**2.2318**	**0.0006**	**90127**	1.8977	0.1701	95953
Vegetation	1,11	**8.0698**	**0.0166**	**90902**	1.3868	0.2027	94250	1.2753	0.2986	91638
Site	11,64	**2.7796**	**0.005**	**93942**	**1.3739**	**0.0184**	**85234**	**2.8065**	**0.0024**	**93127**
Treatment	4,64	**3.2799**	**0.0166**	**95427**	1.1823	0.2218	89803	1.2001	0.3384	95405
VegxTreat	4,64	0.84414	0.5042	95403	0.74004	0.8658	89656	0.68513	0.6413	95284
Entomobryomorpha										
Grassland	5,11	1.7247	0.2112	95876	**2.3356**	**0.0055**	**92441**	2.1277	0.1329	95736
Vegetation	1,11	**13.782**	**0.0031**	**90692**	**3.251**	**0.0153**	**95407**	1.9057	0.1923	93920
Site	11,64	1.3086	0.2406	93765	0.93995	0.5887	88641	0.9197	0.575	92391
Treatment	4,64	1.4863	0.2172	95404	1.4926	0.0891	92063	1.1873	0.3413	94644
VegxTreat	4,64	1.1741	0.3315	95376	1.0832	0.3638	91935	0.78979	0.626	94491
Poduromorpha										
Grassland	5,11	0.50673	0.7648	95846	**1.9101**	**0.0464**	**93395**	1.8502	0.1798	95901
Vegetation	1,11	0.99787	0.3378	90713	0.39846	0.8066	95556	0.57328	0.5243	92831
Site	11,64	**1.981**	**0.0452**	**93916**	**2.4106**	**0.00008**	**89874**	1.6483	0.0656	92826
Treatment	4,64	**3.1969**	**0.0184**	**95451**	0.72646	0.7503	92735	0.55828	0.7673	94931
VegxTreat	4,64	0.81853	0.5201	95441	0.61386	0.8629	92722	1.2358	0.302	94800
Acari										
Grassland	5,11	2.4224	0.1043	95869	**2.5172**	**0.00004**	**88917**	2.1993	0.1271	95757
Vegetation	1,11	3.0325	0.1091	90827	**2.4771**	**0.0071**	**93610**	4.3446	0.0607	90863
Site	11,64	**3.1418**	**0.002**	**93942**	**1.7086**	**0.00004**	**82826**	**3.5677**	**0.0009**	**93905**
Treatment	4,64	1.0753	0.3748	95287	1.3017	0.0896	88286	0.11026	0.9795	95403
VegxTreat	4,64	1.4335	0.2331	95382	0.87046	0.7167	88457	0.54243	0.7112	95404
Prostigmata										
Grassland	5,11	1.9276	0.1687	95933	**2.3054**	**0.0008**	**90616**	2.3627	0.1066	95819
Vegetation	1,11	2.0568	0.1802	90771	1.5127	0.166	94501	0.99945	0.339	90811
Site	11,64	**2.5084**	**0.0105**	**93976**	1.2884	0.0562	85662	**1.9659**	**0.0462**	**93788**
Treatment	4,64	1.8007	0.1383	95450	1.4407	0.0589	90178	0.30858	0.8735	95399
VegxTreat	4,64	0.7644	0.5538	95414	0.87508	0.6658	90232	0.095004	0.9839	95396
Monogynaspida										
Grassland	5,11	0.48632	0.7811	95898	2.0145	0.0716	94521	0.80603	0.5719	95956
Vegetation	1,11	2.8947	0.1172	90696	3.2045	0.0519	96573	**7.0258**	**0.0222**	**90733**
Site	11,64	**2.0762**	**0.035**	**93871**	1.5225	0.062	91341	1.0416	0.4225	93851
Treatment	4,64	0.64107	0.6361	95371	0.55493	0.8365	94091	0.31166	0.8698	95464
VegxTreat	4,64	0.66778	0.6179	95441	0.56961	0.8207	94260	0.12682	0.9735	95495
Oribatida										
Grassland	5,11	2.0268	0.1537	95867	**3.7381**	**0.00001**	**91620**	1.4255	0.2909	95917
Vegetation	1,11	0.59469	0.458	90912	**5.4767**	**0.0002**	**94869**	4.7204	0.0525	90826
Site	11,64	**8.2942**	**0.00001**	**93876**	**2.2963**	**0.00001**	**87306**	**7.6934**	**0.00001**	**93826**
Treatment	4,64	1.1873	0.3274	95456	0.79339	0.7484	91182	0.78894	0.5414	95388
VegxTreat	4,64	2.2573	0.0735	95398	0.85898	0.6635	91026	1.9523	0.1109	95518

Df: degrees of freedom (numerator, denominator). F: pseudo-F value. Perms: unique permutations.

**Fig 2:**
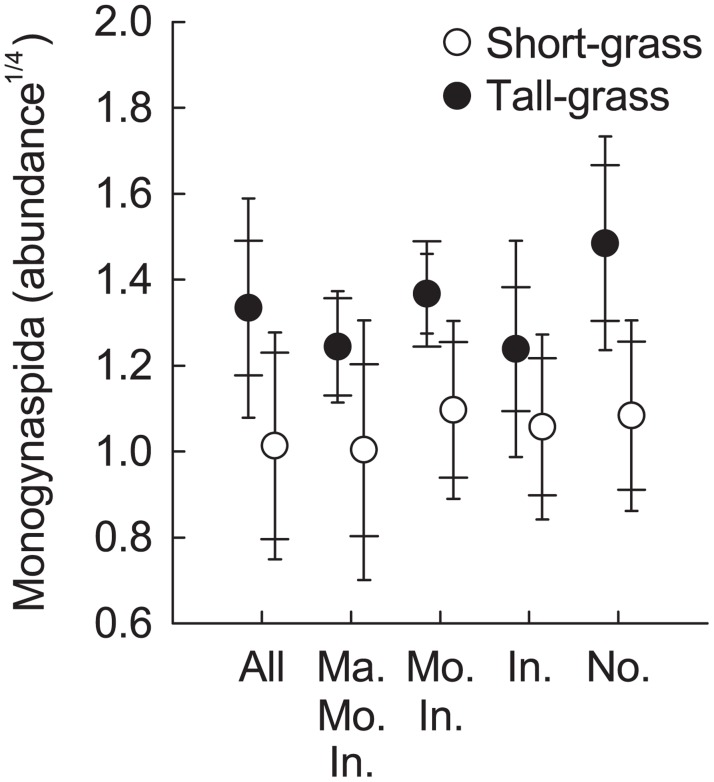
Effects of herbivore exclusion on Monogynaspida abundance in short- and tall-grass vegetation. Treatment abbreviations: “Ma. Mo. In.”- Marmot/Mouse/Invertebrate; “Mo. In.”- Mouse/Invertebrate; “In.”-Invertebrate; “No.”- None. Abundance: number of individuals per soil core summed across three sampling times. Mean ± Cousineau-Morey SE (wide cap—normalised per exclosure setup for treatment comparisons; narrow cap—normalised per grassland for vegetation type comparisons, see [37, 38].

The abundance of Monogynaspida was significantly higher in the tall- than in the short-grass vegetation (Table 1, Fig. 2), while the abundance of none of the other soil microarthropod groups differed among vegetation types. No differences were found in the abundance of any of the groups among the herbivore exclusion treatments (Table 1).

**Fig 3:**
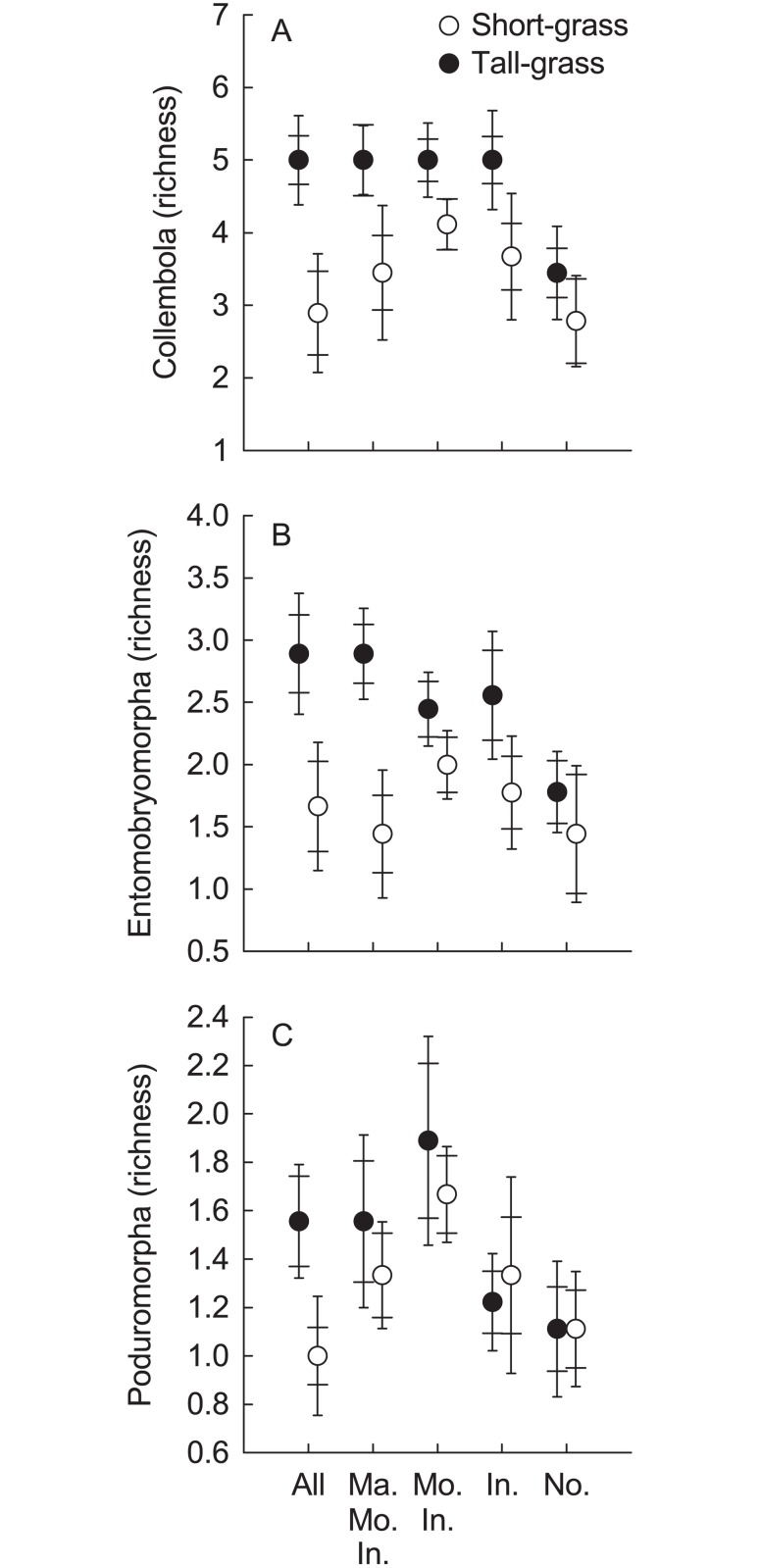
Effects of herbivore exclusion on soil microarthropod richness in short- and tall-grass vegetation. A) Collembola. B) Entomobryomorpha. C) Poduromorpha. Treatment abbreviations: “Ma. Mo. In.”- Marmot/Mouse/Invertebrate; “Mo. In.”- Mouse/Invertebrate; “In.”-Invertebrate; “No.”- None. Richness: number of taxa recorded at least one of the three sampling times. Mean ± Cousineau-Morey SE (wide cap—normalised per exclosure setup for treatment comparisons; narrow cap—normalised per grassland for vegetation type comparisons).

Total springtail richness and richness of the springtail suborder Entomobryomorpha were significantly higher in the tall- than in the short-grass vegetation (Table 1, Fig. 3a,b). There was a significant effect of exclusion treatment on the richness of springtails and the springtail suborder Poduromorpha (Table 1, Fig. 3a,c). After FDR-adjustment, however, none of the pairwise comparisons between different herbivore exclusion treatments was significant for Poduromorpha richness, while Collembola richness was significantly higher in the “Mouse/Invertebrate” than in the “None” treatment. Richness of mites or of any of the mite suborders was not affected by vegetation type or herbivore exclusion treatment (Table 1).

### Effects of vegetation type and herbivore exclusion treatment on vegetation and soil characteristics

**Fig 4:**
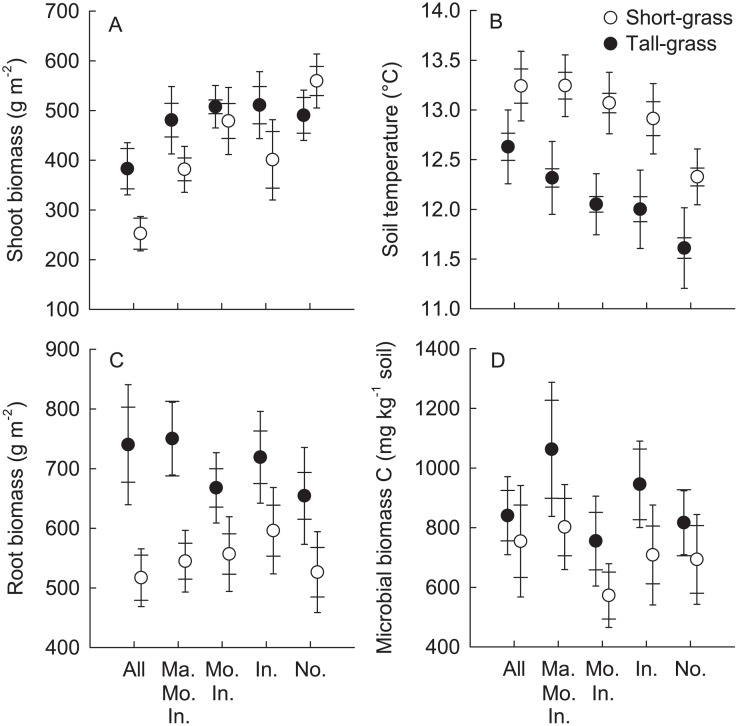
Effects of herbivore exclusion in short- and tall-grass vegetation on A) shoot biomass (g m^-2^), B) soil temperature (°C), C) root biomass (g m^-2^), D) microbial biomass carbon (mg kg^-1^). Treatment abbreviations: “Ma. Mo. In.”- Marmot/Mouse/Invertebrate; “Mo. In.”- Mouse/Invertebrate; “In.”-Invertebrate; “No.”- None. Mean ± Cousineau-Morey SE (wide cap—normalised per exclosure setup for treatment comparisons; narrow cap—normalised per grassland for vegetation type comparisons).

Treatment effects on shoot biomass were more pronounced in the short- than in the tall-grass vegetation (significant vegetation × treatment effect; F_4,64_: 2.66, *P*: 0.040; Fig. 4a). In the short-grass the “All” plots had significantly lower shoot biomass than all other treatments except the “Invertebrate” treatment, indicating an increased consumption by invertebrates if the other herbivores were excluded. Shoot biomass was also significantly higher in the “None” treatment than in the “Marmot/Mouse/Invertebrate” and “Mouse/Invertebrate” treatment. In the tall-grass vegetation, shoot biomass was only significantly higher in the “Mouse/Invertebrate” treatment than in the “All” treatment. Soil temperature was higher in the short- than in the tall-grass vegetation (F_1,11_: 7.98, *P*: 0.047) and significantly differed between the herbivore exclusion treatments (F_4,64_: 17.75, *P <* 0.0001, Fig. 4b). It was significantly lower in the “None” plots than in other treatments in both vegetation types. The “All” plots had a significantly higher soil temperature than all the other plots except the “Marmot/Mouse/Invertebrate” plots. Root biomass was not affected by the progressive exclusion of herbivores, but was significantly higher in tall- than in short-grass sites (F_1,11_: 9.80, *P*: 0.0093; Fig. 4c). Soil microbial biomass C was unaffected by the herbivore exclusion treatment, but was significantly higher in the tall- than in the short-grass vegetation (F_1,11_: 4.85, *P*: 0.050; Fig. 4d). Mineral and organic soil C and N concentrations and C/N ratios as well as soil moisture did not significantly differ between vegetation types or exclusion treatments (all *P*>0.05).

**Fig 5:**
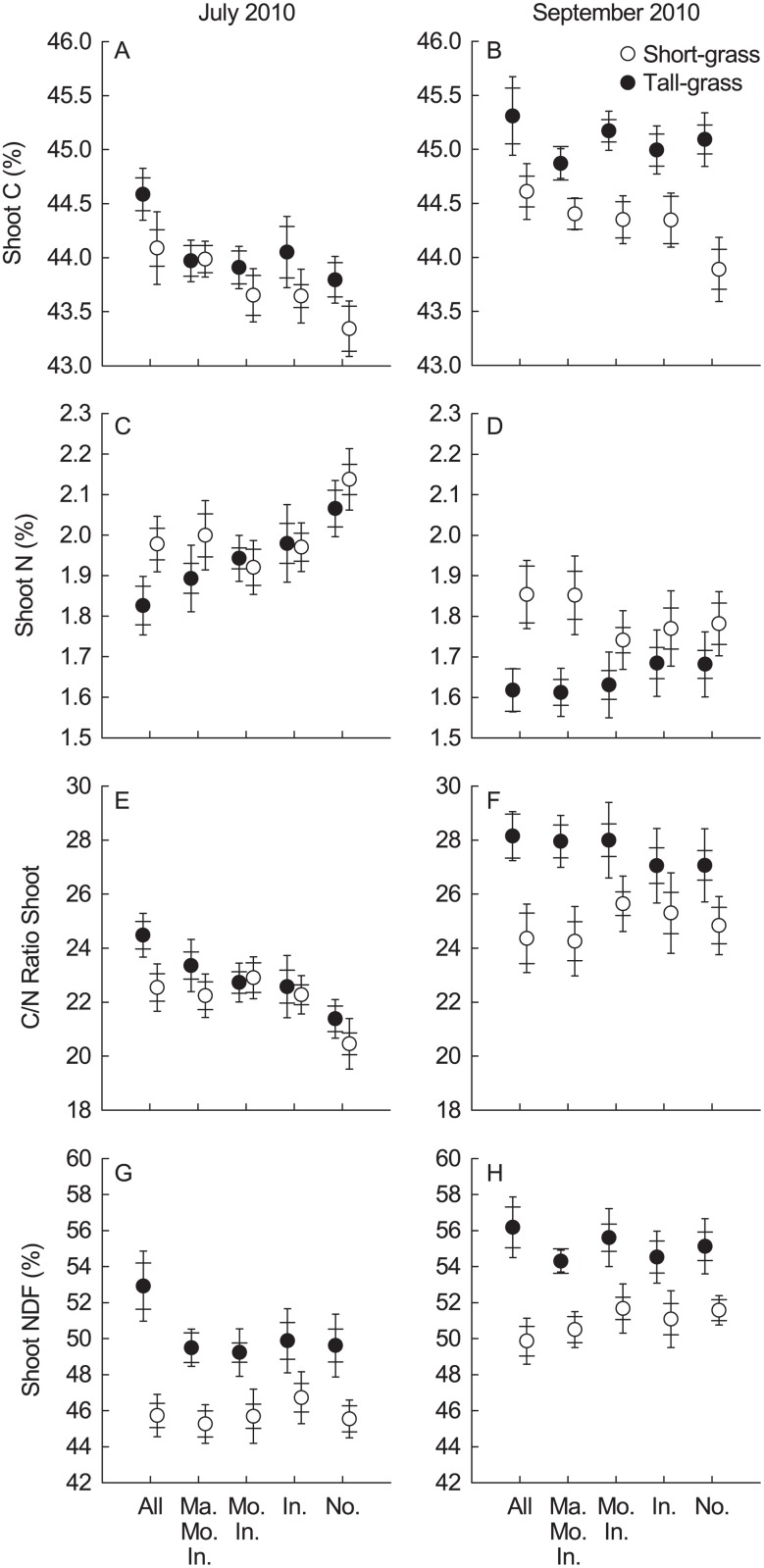
Effects of herbivore exclusion in short- and tall-grass vegetation on A,B) shoot C content (%), C,D) shoot N content (%), E,F) shoot C/N ratio, G,H) Shoot NDF (%). Left panels: July 2010, right panels: September 2010. Treatment abbreviations: “Ma. Mo. In.”- Marmot/Mouse/Invertebrate; “Mo. In.”- Mouse/Invertebrate; “In.”-Invertebrate; “No.”- None. Mean ± Cousineau-Morey SE (wide cap—normalised per exclosure setup for treatment comparisons; narrow cap—normalised per grassland for vegetation type comparisons).

Peak biomass shoot C content differed between herbivore exclusion treatments (F_4,64_: 5.79, *P*: 0.0003, Fig. 5a), with the vegetation in the “All” plots having a significantly higher percentage of C than all other treatments. By September of the same year this treatment effect was no longer apparent, but now C content was significantly higher in the tall- than in the short-grass vegetation (F_1,11_: 35.10, *P <* 0.0001, Fig. 5b). The peak biomass shoot N content (F_4,64_: 6.79, *P <* 0.0001, Fig. 5c) and C/N ratio (F_4,64_: 7.74, *P <* 0.0001, Fig. 5e) also differed between treatments: the vegetation in the “None” plots had a significantly higher percentage of N and lower C/N ratio than that in all other plots. By September, these treatment effects again shifted to a difference between vegetation types, with significantly higher N content (F_1,11_: 6.08, *P*: 0.032, Fig. 5d) and lower C/N ratio (F_1,11_: 7.04, *P*: 0.023, Fig. 5f) in the short-grass vegetation. The concentration of NDF was significantly higher in the tall- than in the short-grass vegetation, and this remained consistent over the season (July: F_1,11_: 14.22, *P*: 0.0034, Fig. 5g, September: F_1,11_: 16.22, *P*: 0.0017, Fig. 5h).

### Mechanisms of herbivore exclusion treatment and vegetation type effects on soil microarthropod taxonomic groups

Table 2a lists all covariates that were included in initial models of microarthropod community composition, abundance or richness in which the vegetation type effect was significant. Several covariates showed a significant relationship to the richness, abundance or community composition of at least one microarthropod group. However, only July and September NDF content, September C content and soil temperature also made the initial vegetation type effect turn non-significant in several models, implying that soil temperature and plant C and fibre were the only variables explaining differences between short- and tall-grass vegetation in microarthropod communities. September C content explained vegetation type differences in Acari and Entomobryomorpha community composition. It showed a positive correlation with Monogynaspida abundance, which explained the higher abundance of this group in the tall-grass vegetation. July NDF content explained the different Acari and Entomobryomorpha community composition in the tall- compared with the short-grass vegetation. September NDF content explained all observed differences between vegetation types in microarthropod communities, except the difference in Entomobryomorpha richness. As the NDF consists of lignin, cellulose and hemicellulose, all C-based compounds, part of the observed effects of C could be due to a correlation with NDF content. Indeed, all effects of vegetation type on microarthropods explained by shoot C content were also explained by shoot NDF content and not the other way around. Soil temperature explained variation between vegetation types in Acari community composition. The negative relationship between Monogynaspida abundance and soil temperature, moreover, explained their higher abundance in the tall-grass vegetation. None of the covariates explained differences in Collembola richness between treatments, whereas the effect of herbivore exclosures on Poduromorpha richness was mediated by a negative relationship between Poduromorpha richness and July shoot N content (Table 2b).

**Table 2:** Summary of effects of adding covariates to initial models with a significant vegetation type effect (A) or with a significant herbivore exclusion treatment effect (B).

*A) Models with significant vegetation type effect*						
	Community composition			Abundance	Richness	
Covariate	Acari	Oribatida	Entomo-bryomorpha	Mono-gynaspida	Collembola	Entomo-bryomorpha
Shoot biomass	S	S	S	+	+	NS
Root biomass	NS	NS	NS	NS	NS	NS
Shoot C July	NS	NS	NS	NS	NS	+
Shoot C Sept.	S, *	S	S, *	+, *	+	+
Shoot N July	NS	NS	NS	NS	NS	NS
Shoot N Sept.	NS	S	NS	NS	NS	NS
Shoot C/N July	NS	NS	NS	NS	NS	NS
Shoot C/N Sept.	NS	S	NS	NS	NS	NS
NDF July	S, *	S	S, *	NS	+	+
NDF Sept.	S, *	S, *	S, *	+, *	+, *	+
C organic soil	NS	NS	NS	NS	NS	NS
C mineral soil	S	NS	NS	NS	NS	NS
N organic soil	NS	NS	NS	NS	NS	NS
N mineral soil	NS	NS	NS	NS	NS	NS
C/N organic soil	NS	NS	NS	NS	NS	NS
C/N mineral soil	S	NS	NS	NS	NS	NS
Soil moisture	NS	NS	NS	NS	NS	NS
Soil temperature	S, *	S	S	-, *	NS	NS
Microbial biomass	S	NS	NS	+	+	+

*B) Models with significant herbivore exclusion treatment effect*		
	Richness	
Covariate	Collembola	Poduromorpha
Shoot biomass	NS	NS
Root biomass	NS	NS
Shoot C July.	NS	NS
Shoot C Sept.	NS	NS
Shoot N July	NS	-, *
Shoot N Sept.	NS	NS
Shoot C/N July	NS	+
Shoot C/N Sept.	NS	+
NDF July	NS	NS
NDF Sept.	NS	NS
C organic soil	NS	NS
C mineral soil	NS	NS
N organic soil	NS	NS
N mineral soil	NS	NS
C/N organic soil	NS	NS
C/N mineral soil	NS	NS
Soil moisture	NS	NS
Soil temperature	NS	NS
Microbial biomass	NS	NS

For each covariate a separate model was run. NS: non-significant (*P* > 0.05). S: significant (P ≤ 0.05) with +: positive relationship,-: negative relationship, *: inclusion of the significant covariate rendered the original vegetation type or herbivore exclusion treatment effect non-significant.

## Discussion

Contrary to our expectations (H1, H2, H3, H4), excluding aboveground herbivores of different size classes had limited effects on soil Acari and Collembola communities. However, these communities strongly differed between the two vegetation types as we hypothesised (H5). In contrast to our hypotheses, the observed effects of the aboveground herbivore exclusions were not explained by the concurrent changes in shoot biomass and soil temperature (H2) and neither were differences between vegetation types explained by root and microbial biomass (H5). However, most differences in soil microarthropod communities between vegetation types or herbivore exclusion treatments were explained by the nutrient or fibre content of the vegetation the year before sampling, which serves as a proxy for the quality of litter. These changes in litter quality could directly have affected soil microarthropods feeding on detritus and indirectly influenced others feeding on soil microorganisms such as fungi. Soil temperature also played a role, as it explained some of the differences in microarthropod communities between vegetation types.

### Effects of herbivore exclusion treatment on soil microarthropod taxonomic groups

Several studies have investigated the effects of a single functional group of herbivores on soil microarthropods (see introduction), but the effects of multiple interacting species that make up the entire aboveground herbivore community remain to our knowledge untested. Contrary to H1, excluding the largest herbivores, the ungulates, did not alter abundance, richness or community structure of any of the microarthropod groups in our study. This contrasts several studies conducted in meadow systems where effects of domestic ungulates on soil microarthropods were evident and mostly caused by disturbance [14, 17, 39]. Lessard et al. [40] demonstrated a negative effect of white-tailed deer on soil microarthropod richness in the Great Smoky Mountains National Park, Tennessee (USA), and attributed this to a decrease in litter quantity. This either means that in our subalpine grasslands excluding ungulates does not reduce disturbance enough to elicit a measurable response of the soil microarthropod communities, that smaller herbivores somehow compensate for the absence of ungulates or that more time than three growing seasons is needed before any changes become apparent. Firstly, densities of red deer and chamois in the SNP are indeed considerably lower than those typical for domestic ungulates. This could explain why removing them did not have an impact on the soil microarthropod community, whereas excluding large domestic ungulates from meadow systems did. Secondly, these subalpine soils may need considerably more time than three growing seasons to recover from soil compaction or changes in the litter layer caused by ungulates. Lessard et al. [40], for example, used deer exclosures that had been in place for 13 years at the time of sampling. They reported densities of 35 deer per km^2^, which is similar to the densities observed in our system (5 year average of between 22 and 37 ungulates per km^2^ depending on location). This suggests that our lack of responses is more likely due to the shorter time frame or the compensatory grazing by smaller herbivores than to insufficient ungulate densities.

In contrast to H1, the exclusion of medium-sized burrowing herbivores in addition to ungulates did not result in any changes in soil microarthropod community structure, abundance or richness either. Only Poduropmorpha richness was significantly lower in control plots than in plots excluding medium-sized herbivores, but this difference was no longer significant after correcting for multiple comparisons. Ukabi et al. [22] showed a negative effect of porcupine burrowing on soil microarthropods in the Negev desert, but their study specifically compared microarthropod communities in burrows and control plots. In contrast, burrowing activities by marmots in our plots are rather limited so that excluding them may not have altered disturbance enough for the soil microarthropods to respond. Alternatively, the timespan of our study may have been too short for the soils to recover from burrowing disturbance or other alterations to the system by medium-sized mammals.

We detected a reduction in overall Collembola richness, when the entire aboveground herbivore assemblage was excluded (“None” plots), compared with when only ungulates and medium-sized herbivores were excluded (“Mouse/Invertebrate” plots). The lower Collembola richness in invertebrate exclosures is in line with our hypothesis that soil microarthropods benefit from aboveground invertebrate herbivores (H4). However, this reduction did not differ between the two vegetation types, in disagreement with H3. We are not aware of any study that assessed the effects of small rodents on soil microarthropods or of invertebrate herbivores on soil microarthropod richness. We can therefore only compare our results to microcosm studies of single soil microarthropod species’ responses to invertebrate herbivores. Bradford et al. [41] demonstrated that caterpillar herbivory caused changes in aboveground leaf litter quality with transient negative effects on Collembola abundance in a soil microcosm experiment. In other microcosm studies positive effects of shoot-feeding aphids on springtail densities have been attributed to increased root exudation, stimulation of microbial biomass by honeydew or increases in dead root biomass [42–44]. Thus, these studies indicate that effects of aboveground invertebrate herbivores on soil microarthropod populations can be positive or negative. Our exclusion treatments did not change the abundance of any of the soil microarthropod groups, therefore our results contrast those of the mentioned microcosm studies. It is possible that positive and negative effects cancelled each other out in the field. Yet, the lower springtail richness in the “None” treatment, together with the fact that both springtail community structure and abundance were not significantly affected, suggests that rare taxa with a limited contribution to overall community structure disappeared in favour of more common ones when all herbivores were excluded. Also note that the cited microcosm studies only manipulated herbivory by invertebrates, similar to comparing the “Invertebrate” and “None” treatments. In our system either the effect of the invertebrates interacted in some way with the presence of small mammals, or the additive effects of both small mammals and invertebrates were needed to cause a significant change in springtail richness. Petersen et al. [16] found that certain litter dwelling Collembola species benefited from grazing, albeit by livestock, and suggested this was due to reduced shoot biomass and increased soil temperature. However, this logical assumption (H3) was not supported by our mechanistic analyses, as neither shoot biomass nor soil temperature explained the treatment effect on springtail richness, while the negative relationship with shoot N content at peak biomass did in the case of Poduromorpha richness. Although litter quality has been suggested as a link between aboveground herbivores and soil microarthropod diversity [14], we are not aware of any study explicitly testing this. The positive influence of litter quality on litter-dwelling microarthropods is well established [45], whereas soil-dwelling microarthropods, however, generally do not respond to litter quality [46–49]. Yet the decrease in Poduromorpha richness was coupled with the increase of shoot N in our study. This increase could be a direct consequence of the exclusion of sap-sucking and leaf-chewing insects respectively feeding on N-rich phloem and the most N-rich plant parts. The more N-rich litter returned to the soil may have resulted in a shift from fungal to bacterial dominance [50, 51], for example excluding the fungi that certain Poduromorpha depend upon [52, 53].

### Effects of vegetation type on soil microarthropod taxonomic groups

As predicted, soil microarthropod communities differed significantly between the two vegetation types (H5), with higher overall Collembola and Entomobryomorpha richness and higher Monogynaspida abundance in the tall-grass vegetation. The community composition of Entomobryomorpha, Acari and Oribatida also differed between vegetation types. Historical grazing intensity, mediated by changes in vegetation type, thus had a profound impact on soil microarthropod communities. This shows that over longer timescales, ungulates can indeed alter these communities, corroborating the idea that the microarthropods might have reacted to our ungulate exclusions if given more time. For example, the hard-bodied oribatid mites often have multi-year lifespans [17, 21, 45], which could explain why their communities did not react to three growing seasons of herbivore exclusion treatments, but differed between the vegetation types. Even though root and microbial biomass were higher in the tall-grass vegetation, these differences were-in contrast to H5- not responsible for the more abundant and diverse microarthropod community. Different measures of plant quality, however, explained differences in richness, abundance and community composition of several microarthropod groups between the two vegetation types. Plant material collected in the tall-grass vegetation had a higher fibre content. Fibre complexes are mostly decomposed by fungi rather than by bacteria [54, 55]. A higher fungal dominance could thus have occurred in the tall-grass vegetation, explaining the higher microarthropod abundance and springtail richness, as many mites and springtails are known to feed on fungi [53, 56]. The higher C content of the vegetation may furthermore represent a higher proportion of organic compounds in the litter entering the soil, on which many microarthropods can also feed directly [53, 57]. Our results thus suggest that in the tall-grass vegetation increased resource quality, either microbial or detrital, stimulated microarthropod communities. The fact that the largely predatory Monogynaspida reached higher densities in the tall-grass than in the short-grass vegetation further suggests that the roughly 70 years since vegetation types started diverging allowed enough time for this increased resource quality to transfer energy up to the higher trophic levels. These are now potentially controlling lower trophic levels, such as springtails and Prostigmata, whose abundance did not differ between vegetation types. By selectively feeding on dominant taxa, the predators could have contributed to the increase in springtail richness by reducing competitive exclusion as suggested by Cole et al. [58]. Several authors have pointed at the importance of bottom-up forces in structuring soil microarthropod communities [21, 25, 58]. Yet there is still considerable debate, as other studies found no or idiosyncratic relationships with resource availability [59, 60] or suggested top-down controls to be important [61]. Some authors tested relationships between resources, such as microbial or root biomass, and soil microarthropods [25, 58, 59], whereas others merely assumed such relationships to explain effects of grazing or other manipulations on soil microarthropods [15, 60]. Our results show that such assumptions can be misleading. For example, root biomass was higher in the tall- than in the short-grass vegetation, but as a covariate it did not relate to any of the microarthropod groups with higher richness or abundance in the tall-grass vegetation. Microbial biomass was also higher in the tall-grass vegetation and related positively across samples with Monogynaspida abundance and Entomobryomorpha and springtail richness, which were higher in the tall-grass vegetation as well. Nonetheless, our analyses indicated that plant quality and soil temperature, not microbial biomass explained the differences in soil microarthropod communities between vegetation types. The lower soil temperature in the tall-grass vegetation did not explain the higher Collembola richness. However, it explained the different Acari community composition and the higher abundance of Monogynaspida in this vegetation type, in contrast to our hypothesis. In a meta-analysis, Blankinship et al. [62] showed that soil biota are more likely to respond negatively to warming in colder climates. Given that the subalpine meadows are exposed to high levels of solar radiation, the lower amount of shade in the short-grass vegetation may have allowed the soil temperature to reach a harmful level for these microarthropods that are adapted to the cold.

In summary, four of our five hypotheses derived from the literature were not or just partly confirmed by the response patterns of soil microarthropod communities to aboveground herbivores found in our experiment. This could simply mean that results from most former studies dealing with agro-pastoral systems or microcosm experiments may not be directly applicable to ecosystems such as the wilderness area of our experiment. The expected short-term effects of large ungulates reported from agro-pastoral systems could, for example, not be found in our grassland sites grazed by wild ungulates. However, long-term effects of large grazers became apparent through changes in the structural and chemical characteristics of the vegetation. In contrast, small herbivores were found to have short-term effects on the soil microarthropod communities, suggesting that soil arthropods benefit from aboveground invertebrate herbivores. These functionally important small herbivores should, thus, not be overlooked in future studies.

## Supporting Information

S1 Table Soil microarthropod abundances (summed across three sampling dates). (XLSX)
